# Oral presentation assessment and image reading behaviour on brain computed tomography reading in novice clinical learners: an eye-tracking study

**DOI:** 10.1186/s12909-022-03795-9

**Published:** 2022-10-25

**Authors:** Chi-Hung Liu, June Hung, Chun-Wei Chang, John J. H. Lin, Elaine Shinwei Huang, Shu-Ling Wang, Li-Ang Lee, Cheng-Ting Hsiao, Pi-Shan Sung, Yi-Ping Chao, Yeu-Jhy Chang

**Affiliations:** 1 Department of Neurology, Linkou Medical Center, Chang Gung Memorial Hospital, Taoyuan, Taiwan; 2 School of Medicine, College of Medicine, Chang Gung University, Taoyuan, Taiwan; 3 Division of Medical Education, Graduate Institute of Clinical Medical Sciences, College of Medicine, Chang Gung University, Taoyuan, Taiwan; 4 Institute of Health Policy and Management, College of Public Health, National Taiwan University, Taipei, Taiwan; 5 Graduate Institute of Science Education, National Taiwan Normal University, No. 88, Ting-Jou Rd., Sec. 4, Taipei City, Taiwan; 6 Graduate Institute of Digital Learning and Education, National Taiwan University of Science and Technology, Taipei, Taiwan; 7 Department of Otorhinolaryngology-Head and Neck Surgery, Linkou Main Branch, Chang Gung Memorial Hospital, Taoyuan, Taiwan; 8 Institute of Brain Science, National Yang Ming Chiao Tung University, Taipei, Taiwan; 9 Department of Emergency Medicine, Chang Gung Memorial Hospital, Chiayi, Taiwan; 10 Chang Gung Medical Education Research Centre, Taoyuan, Taiwan; 11 Department of Neurology, College of Medicine, National Cheng Kung University Hospital, National Cheng Kung University, Tainan, Taiwan; 12 Department of Computer Science and Information Engineering, Chang Gung University, Taoyuan, Taiwan; 13 Department of Biomedical Engineering, Chang Gung University, Taoyuan, Taiwan

**Keywords:** Assessment, Eye-tracking, Brain CT education, Oral presentation, Reading behaviour

## Abstract

**Background:**

To study whether oral presentation (OP) assessment could reflect the novice learners’ interpretation skills and reading behaviour on brain computed tomography (CT) reading.

**Methods:**

Eighty fifth-year medical students were recruited, received a 2-hour interactive workshop on how to read brain CT, and were assigned to read two brain CT images before and after instruction. We evaluated their image reading behaviour in terms of overall OP post-test rating, the lesion identification, and competency in systematic image reading after instruction. Students’ reading behaviour in searching for the target lesions were recorded by the eye-tracking technique and were used to validate the accuracy of lesion reports. Statistical analyses, including lag sequential analysis (LSA), linear mixed models, and transition entropy (TE) were conducted to reveal temporal relations and spatial complexity of systematic image reading from the eye movement perspective.

**Results:**

The overall OP ratings [pre-test vs. post-test: 0 vs. 1 in case 1, 0 vs. 1 in case 2, p < 0.001] improved after instruction. Both the scores of systematic OP ratings [0 vs.1 in both cases, p < 0.001] and eye-tracking studies (Case 1: 3.42 ± 0.62 and 3.67 ± 0.37 in TE, p = 0.001; Case 2: 3.42 ± 0.76 and 3.75 ± 0.37 in TE, p = 0.002) showed that the image reading behaviour changed before and after instruction. The results of linear mixed models suggested a significant interaction between instruction and area of interests for case 1 (p < 0.001) and case 2 (p = 0.004). Visual attention to the target lesions in the case 1 assessed by dwell time were 506.50 ± 509.06 and 374.38 ± 464.68 milliseconds before and after instruction (p = 0.02). However, the dwell times in the case 2, the fixation counts and the frequencies of accurate lesion diagnoses in both cases did not change after instruction.

**Conclusion:**

Our results showed OP performance may change concurrently with the medical students’ reading behaviour on brain CT after a structured instruction.

**Supplementary Information:**

The online version contains supplementary material available at 10.1186/s12909-022-03795-9.

## Introduction

Stroke is one of the leading causes of death and disability in Taiwan and the second.

leading cause worldwide [1]. The phrase “time is brain” is a reminder that acute stroke treatment should be completed as soon as possible. Compared to community onset stroke, patients of hospital onset stroke had longer times from symptom onset to non-contrast brain computed tomography (CT), and more frequently had delayed management [2]. Therefore, brain image reading techniques are an important topic during clinical training. Not only doctors of emergency, radiology, or neurology departments but also primary physicians should all be able to master these image reading techniques in order to propose or initiate adequate treatment plans. Education in medical image reading should focus on the development of these main skills.

Several methods have been proposed to assess learners’ image interpretation, including direct observation skills [3], standardized interpretation training [4], and having an expert double check [5]. Although reporting an accurate diagnosis is a common method to assess a learners’ performance by means of oral reporting or simple multiple-choice questions, the process of a learners’ clinical reasoning may not be easily revealed through these tools unless they are well designed [6]. Reporting in a single best answer manner may give a false impression of the learners’ competence [7]. Oral presentation (OP) maybe limited by the lack of standardisation, making it difficult to understand whether trainees use standardized reading sequences during their presentation. In addition, it is not known whether advances in OP are parallel to changes in image reading behaviours. The global-focal search pattern requires a higher cognitive demand and could be the objective of image reading training [8]. An assessment tool to evaluate whether the learners look at potentially relevant locations or scan the image to locate additional abnormalities that are not salient enough to be found [9, 10] are important for novice learners.

Eye-tracking technique has been extensively applied in medical education [11]. That is, eye tracking could be used to identify patterns of visual attention while performing tasks. Visual disciplines such as radiology could benefit from a better understanding of how experts adapt eye movements in a reading task. Eye movement measurements may reflect connections between cognitive processes and the visual attention [12], and could be suitable to correlate the image reading behaviour to oral reporting performance. Therefore, the aim of this study was to investigate whether OP assessments could reflect the interpretation skills and reading behaviour on brain CT of novice clinical learners after a structured instruction using an eye-tracking technique.

## Methods

### Participant enrolment and study protocol

**Fig. 1:**
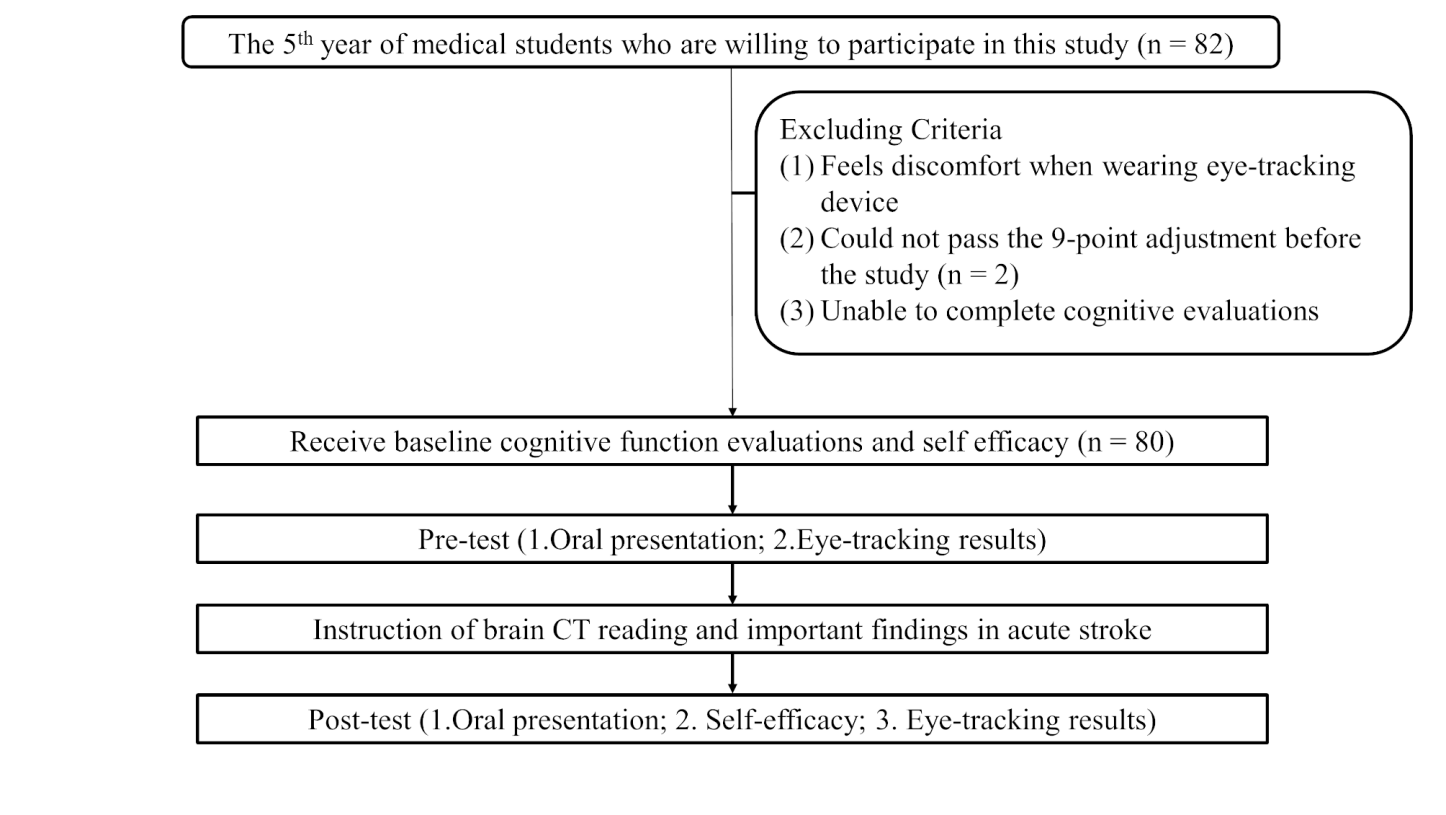
Flow chart of the study protocols

From August 2017 to July 2019, we recruited fifth year medical students from Chang Gung University who had never received organized brain CT training programs and were defined as novice clinical learners. Students who were unwilling to participate in this study, felt discomfort when wearing eye-tracking devices, and those unable to pass the 9-point adjustment of the eye-tracking device, which was used to ensure the accuracy of the eye detection and gaze-point calculation, were excluded from the study (Fig. 1). Participants’ previous experience in neurology lectures and clinical rotation were recorded. In addition, the health status of their eyes was also recorded. The participants received instruction on how to interpret brain CT images. OP and eye-tracking assessments were performed before (pre-test) and after (post-test) the instruction. A self-efficacy questionnaire regarding brain CT interpretation skills was also completed. This study was carried out in accordance with ethical guidelines of Chang Gung Memorial hospital and was also in accordance with Declaration of Helsinki. The study was approved by the Ethics Institutional Review Board of Chang Gung Memorial Hospital (201601984B0), and all participants signed written informed consent.

### Instruction on how to read brain CT images

The participants were divided into eight small groups. We held a 2-hour interactive workshop regarding brain CT reading for each group, with each group containing 10 students. All the workshops were held by the same lecturer. The milestone of acute stroke image reading may include reading images systematically, identifying common lesions, identifying multiple and hidden lesions, and integrating findings from multiple image modalities based on the experience and prior knowledge of the learners. The objective of the workshop for medical students in this study was to guide the learners to read the brain CT images in a systematic manner, rather than identifying the pathology of the lesions. A 90-minute lecture was given first to demonstrate the skills of how to read brain CT images, followed by a 30-minute interactive brain CT interpretation session. All the students were asked to report the findings of selected brain CT images, and immediate feedback was given by the lecturer. The contents of the teaching material and the essential items for students were formulated using a web-based Delphi process[13].

### Eye-tracking evaluation

**Fig. 2:**
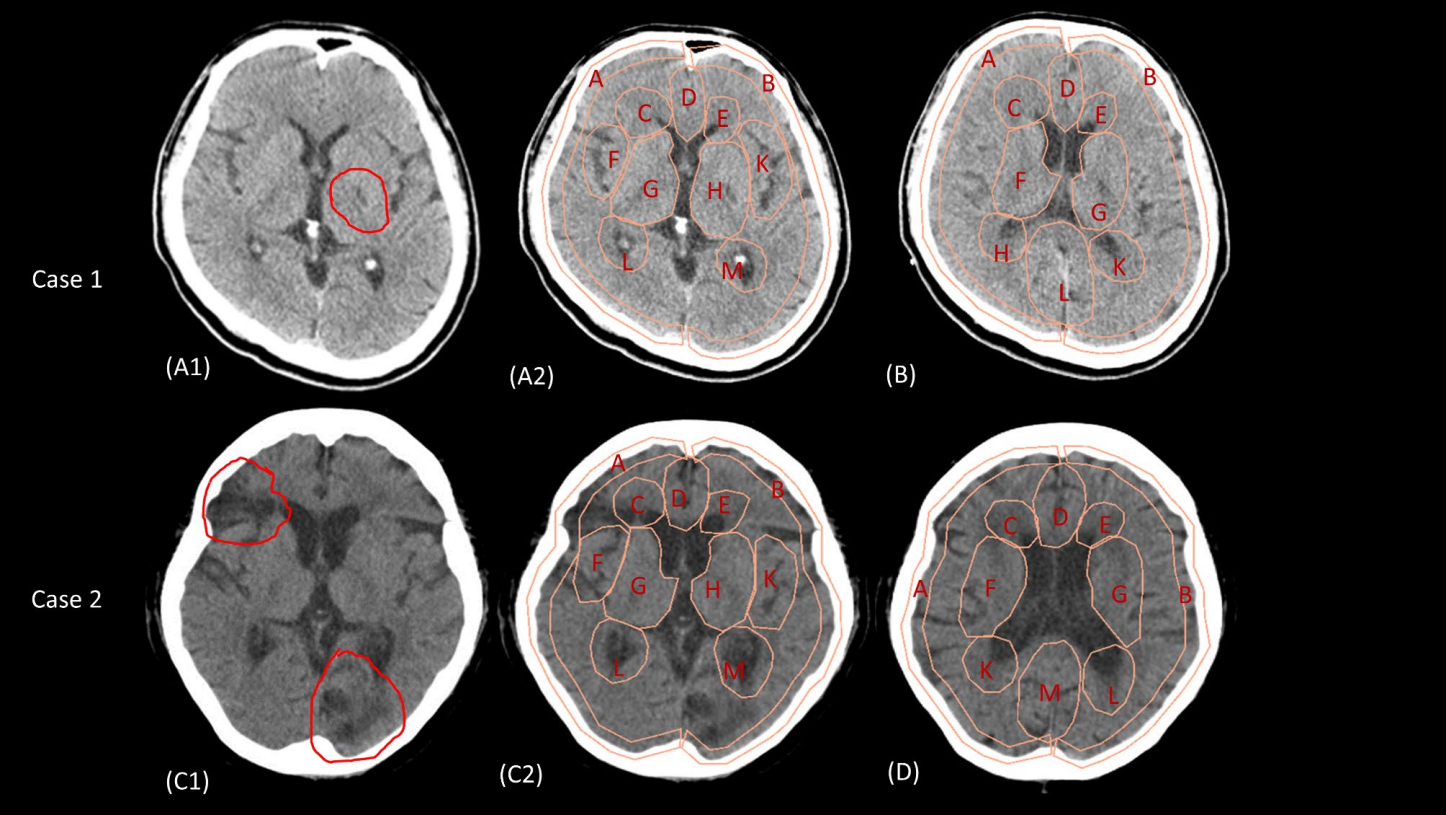
Areas of interest (AOIs) of the selected figures in each case Figure A and B were used in the first case, while Figures C and D were used in the second case. Figure A1 and C1 show the AOIs used for the analyses of dwell time and fixation counts. Figures A2, B, C2, and D demonstrate the AOIs used for the analyses of lag sequential analysis and transition entropy

The students’ visual attention while performing tasks was recorded using an Eye-Link 1000 eye tracker [14], which is a desktop-mounted eye tracker with a sampling rate of 1000 Hz. To increase the precision of data collection, a head rest was used. The eye tracker was placed 15 cm away from the screen, and the distance from the camera to the students was set to about 60 cm. The average accuracy of the eye tracker was between 0.25° and 0.5°, and the operational range was between 45 and 75 cm. The CT slides were shown on a 22-inch monitor with a resolution of 1,028 × 768 pixels based on the EL1000_InstallationGuide_version1.52 (SR Research Ltd., Canada). To make the presentation comparable to that of actual CT slides in clinical practice, we adjusted the resolution of the CT scan and asked several domain experts to confirm the results. The experiment was designed using Experiment Builder version 2.3.38 (SR Research Ltd., Canada). All the students must pass the 9-point calibration before the imaging trials began. The students needed to complete two cases. Each case included two brain images from the same diseased patient. The first case (Fig. 2 A and 2B) suggested a patient with a left internal capsule lacunar infarction (single lesion), and the second case (Fig. 2 C and 2D) illustrated another patient with right frontal and left occipital lesions (multiple lesions). The students had 10 s to read any one of the two images in each case, and had another 30 s to complete their OP (Figure S1). The students were requested to read brain CT images in a systematic way and identify the lesions if feasible.

### Self-efficacy and ratings of OP

The OP of each student was recorded, labelled with serial numbers, and rated by an expert. A grading system (grade from 0 to 4), consisting of the accuracy of lesion reporting and competency in systematic image reading, was used to rate the OP performance. Zero points indicated that the student failed to correctly report the lesion and was unable to interpret the brain image in a systematic manner, while 4 points suggested that the student could correctly report the lesion and was able to interpret the brain image in a systematic manner. Details of the operating definition of each point are listed in Table S1 and S2.

The students were also asked to report their self-efficacy regarding brain CT reading. Self-efficacy is defined as a person’s belief in their ability to execute a series of actions to produce designated levels of performance. The Chinese version of self-efficacy evaluation was used in this study, which has been validated in studies conducted by SLW (Table S3) [15].

### Outcomes

The primary outcome was the overall OP rating after instruction. The secondary outcomes included the lesion identification, and competency in systematic image reading after instruction. Regarding lesion identification, we recorded the accuracy of lesion reporting from OP and the students’ visual attention to the target lesions by recording dwell time (DT) and fixation counts (FCs) in the eye-tracking study [11]. DT refers to the total gaze duration for each area of interest (AOI), and FC refers to the number of fixations with respect to each AOI (Fig. 2A1 and 2B1 for case 1 and 2)[16, 17]. DT and FCs are pervasively in measuring duration and frequency of visual attention. For example, R Koh et al. investigated attentional strategies of novice nurses during surgeries in terms of DT [18]. As for competency in systematic image reading, we recorded the scores of systematic OP ratings and the students’ temporal relationship with reading behaviour using lag sequential analysis (LSA), linear mixed models (LMMs), and transition entropy (TE) in the eye-tracking study. Self-efficacy was also a secondary outcome.

### Statistical analyses

Data were analysed using the SPSS version 22.0 (SPSS, Chicago, IL, USA). Parameters are represented as mean ± standard deviation, n (%), or median (25th, 75th quartile). Categorical variables were compared using the McNemar test. Nonparametric data, including OP rating and self-efficacy scores, were analysed using the Wilcoxon signed-rank test. Continuous data including the eye-tracking results were compared using the Wilcoxon signed-rank tests. Eye-tracking data were pre-processed by merging fixations in the same AOI using Data Viewer (2020, SR-Research) and then imported the AOI report into SAS (2008, SAS Institute, Cary, NC, USA) for further analysis. Given the built-in Four-Stage Fixation Cleaning algorithm was performed by default, the duration thresholds for the first three stages were 80, 40, and 140 milliseconds, respectively. In the fourth stage, the minimum and maximum duration were 140 and 800 milliseconds. The distance between two fixation points (the distance threshold) is within 1°. A SAS program that could process complex off-line and on-line data of eye movement was used [19]. The program consists of three steps. First, online data and offline data were integrated. The online data were the responses collected while the students were reading the CT images. Such data could be helpful when evaluating the students’ attention patterns, and reflected the underlying cognitive processes. In contrast, the offline data involved the students’ subjective assessments. Analysis of the offline data provide insights into the students’ characteristics, especially those related to the task. Second, data were reconstructed to meet the standard of different statistical analyses. Third, statistical analyses were conducted according to the design of this study.

Visual attention was measured in terms of eye movement, including DT and FC. The reason why DT and FC were used is that they have shown potential in detecting cognitive processes during visual search, especially in problem-solving analysis [20, 21]. In contrast to measuring mean tendency of visual attention, the LSA [22], LMMs, and TE were used to explore the distribution of attention on the AOIs, as well as temporal relationships among AOIs (Fig. 2A2/C and 2B2/D for case 1 and 2) [23, 24]. Regarding the rationale for using LSA, LSA could identify significant attentional shift among AOIs form a chronological perspective. That is, LSA helps us to evaluate whether the participants could perform systematic viewing. To perform LSA, FCs in each AOI were aggregated and sorted in a chronological order. The significance of each transition from one AOI to another was calculated in terms of z-score and p value. A Z-score ≥1.96 (p < 0.05) indicated a significant attentional shift between two AOIs. The sequences of attentional shifts were illustrated using transition diagrams. For the LMM, the covariance parameters are estimated using maximal-likelihood methods and the covariance structure in unstructured. The fixed effects include instruction (pre- and post-instruction), while subjects and AOIs were treated as random effects. The LMM examines whether instruction and AOI have effects on DT. The LMM helps to explorer the distribution of DT on the AOIs before and after instruction. In addition to the LMM, the uncertainty of a sequence of eye movements can be scaled in terms of TE [25]. TE was used to evaluate the levels of distribution of visual attention in terms of frequencies of attentional shifts among AOIs. The reason why LMM and TE were employed in the study was that they could be used to examine whether the patterns of visual attention would change after the structured instruction. Specifically, the patterns reflect whether participants could read something important, instead of doing visual search on the CT scan. Following the definition of Hao, Sbert, and Ma [26], TE was defined as:

1 $$\text{T}\text{E}\left(\text{x}\right)= -\sum _{i\in x}p\left(i\right){log}_{2}p\left(i\right)$$

Where x refers to the set of AOIs, and p(i) refers to the probability of attentional shift from AOI I to another AOI. According to Hao et al. [26], low TE indicates that a participant prefers some AOIs over other AOIs. The difference between LSA and TE is that LSA measures local attentional shifts among AOIs, while TE measures global attentional shift among AOIs of a stimulus (a CT scan in this case). TE was calculated based on the frequency of saccade using the ‘Grpstring’ package in R (open-source, GNU General Public License) [27].

## Results

### Baseline characteristics of the enrolled students

Between 2017 and 2019, a total of 82 medical students were initially recruited, two of whom were excluded as they failed to pass the 9-point adjustment before the study. Of the 80 finally enrolled students, 47 (58.8%) were male, and the mean age at enrolment was 23.91 ± 1.33 years. The mean eye-glass prescriptions of their right and left eyes were − 4.11 ± 2.33 and − 3.88 ± 2.36 dioptres, respectively, and the mean prescription to correct astigmatism was 78.41 ± 56.56 dioptres. Sixty-three of the 80 students (78.8%) had rotated to the neurology/radiology ward during their clerkship before study enrolment (Table 1).

**Table 1:** Baseline characteristics of the students

Number	80
Sex (male, %)	47 (58.8%)
Age at enrolment (years old)	23.91 ± 1.33
Eye-glass prescription, right (dioptres)	-4.11 ± 2.33
Eye-glass prescription, left (dioptres)	-3.88 ± 2.36
Prescription of astigmatism	78.41 ± 56.56
Experience of neurology department rotation	63 (78.8%)
History of laser eye surgery (%)	1 (1%)

### Primary outcome

The results, examined by Wilcoxon signed-rank tests, showed that the overall OP rating [Case 1: pre-test vs. post-test, 0 (0,1) vs. 1 (1,2), p < 0.001; Case 2: 0 (0,1) vs. 1 (1,1), p < 0.001] improved after instruction (Table 2).

**Table 2:** Primary and secondary outcomes of the study

				Pre-test		Post-test		p value
**Primary outcome**								
	Overall OP ratings of case 1			0 (0,1)		1 (1,2)		< 0.001*
	Overall OP ratings of case 2			0 (0,1)		1 (1,1)		< 0.001*
**Secondary outcomes**								
	*Lesion identification*							
	Case 1							
		Accuracy of lesion reporting (%)		0 (0%)		1 (1.3%)		1.00
		Dwell time (milliseconds)^§^		506.50 ± 509.06		374.38 ± 464.68		0.02*
		Fixation count^§^		1.98 ± 1.76		1.59 ± 1.91		0.14
	Case 2							
		Accuracy of occipital lesion reporting (%)		11 (13.8%)		13 (16.3%)		0.82
		Accuracy of frontal lesion reporting (%)		7 (8.8%)		11 (13.8%)		0.34
		Dwell time (milliseconds)^#§^		2405.20 ± 1448.11		2518.90 ± 1102.71		0.56
		Fixation count^#§^		8.84 ± 5.18		9.09 ± 3.48		0.72
	*Competency in systematic image reading* Case 1							
		Scores of systematic OP ratings		0 (0,1)		1 (1,2)		< 0.001*
		Transition entropy^§^		3.42 ± 0.62		3.67 ± 0.37		0.001*
	Case 2							
		Scores of systematic OP ratings		0 (0,1)		1 (1,2)		< 0.001*
		Transition entropy^§^		3.42 ± 0.76		3.75 ± 0.37		0.002*
	*Scores of self-efficacies*			35.5 (29,41)		38.5 (32,43)		0.08

^#^summation of both frontal and occipital lesions; ^§^Examined by eye-tracking study;

OP; oral presentation; *p < 0.05

### Secondary outcomes

Regarding the lesion identification, the results of the McNemar tests suggested that the frequencies of accurate lesion reporting did not improve in either case after instruction (Case 1: pre-test vs. post-test, 0% vs. 1.3%, p = 1.00; Case 2: 13.8% vs. 16.3%, p = 0.82 for the occipital lesion, 8.8% vs. 13.8%, p = 0.34 for the frontal lesion). Wilcoxon signed-rank tests were employed to compare eye movements for both cases. In case 1, the pre-test and post-test DTs of the target lesions (Fig. 2 A and 2B) were 506.50 ± 509.06 and 374.38 ± 464.68 milliseconds, respectively (p = 0.02), and the FCs were 1.98 ± 1.76 and 1.59 ± 1.91, respectively (p = 0.54). In case 2, pre-test and post-test total DTs of the two target lesions (Fig. 2 C) were 2405.20 ± 1448.11 and 2518.90 ± 1102.71 milliseconds, respectively (p = 0.40; Table 2), and the FCs were 8.84 ± 5.18 and 9.09 ± 3.48, respectively (p = 0.41).

**Fig. 3:**
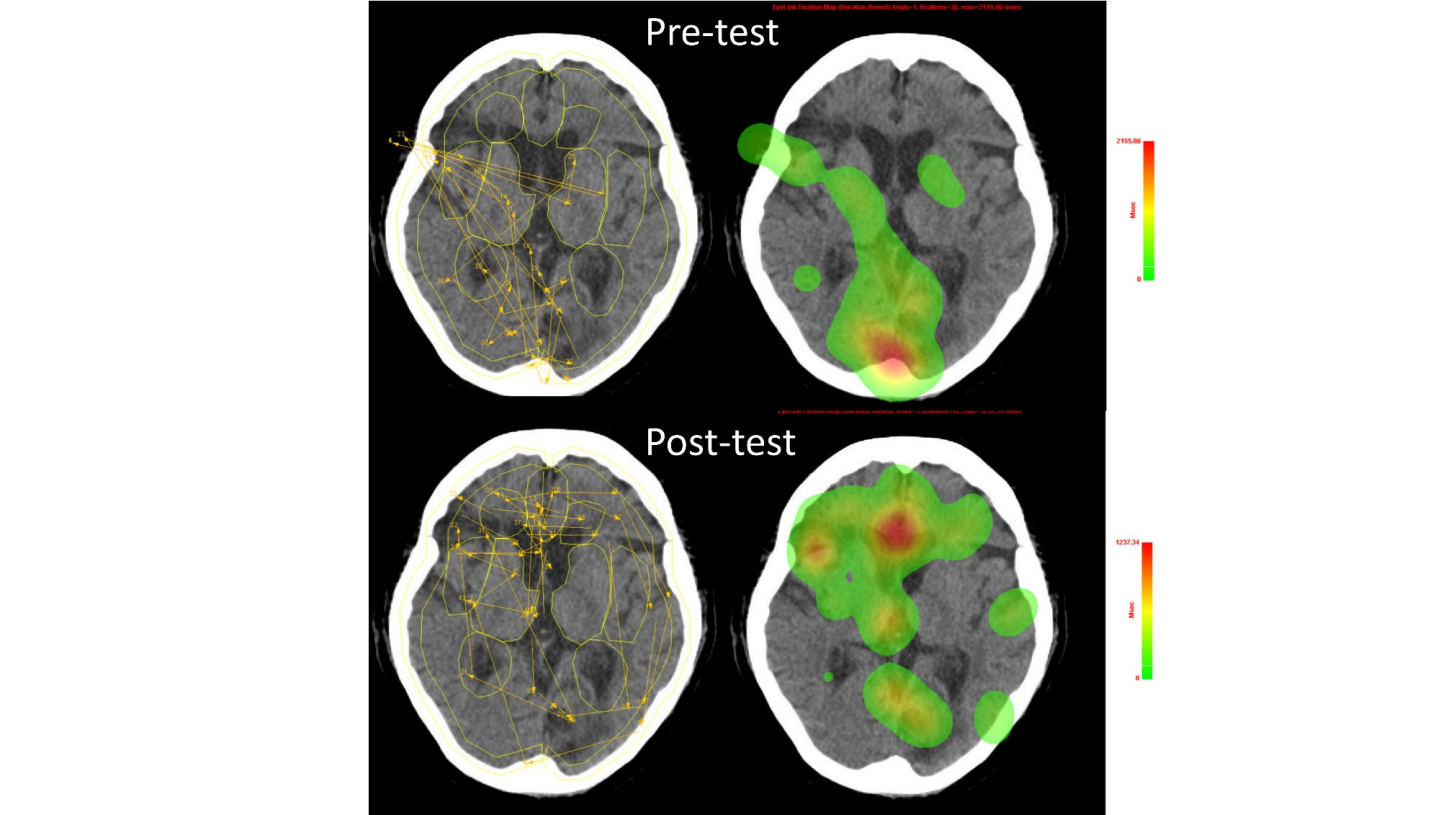
Scan path and heat map before and after instruction in a representative student The upper row shows a deviating reading pattern in the scan path (left) and heat -map (right) before instruction (pre-test). After instruction (post-test), the scan path (lower left) and heat map (lower right) demonstrated a more generalized reading behaviour

**Fig. 4:**
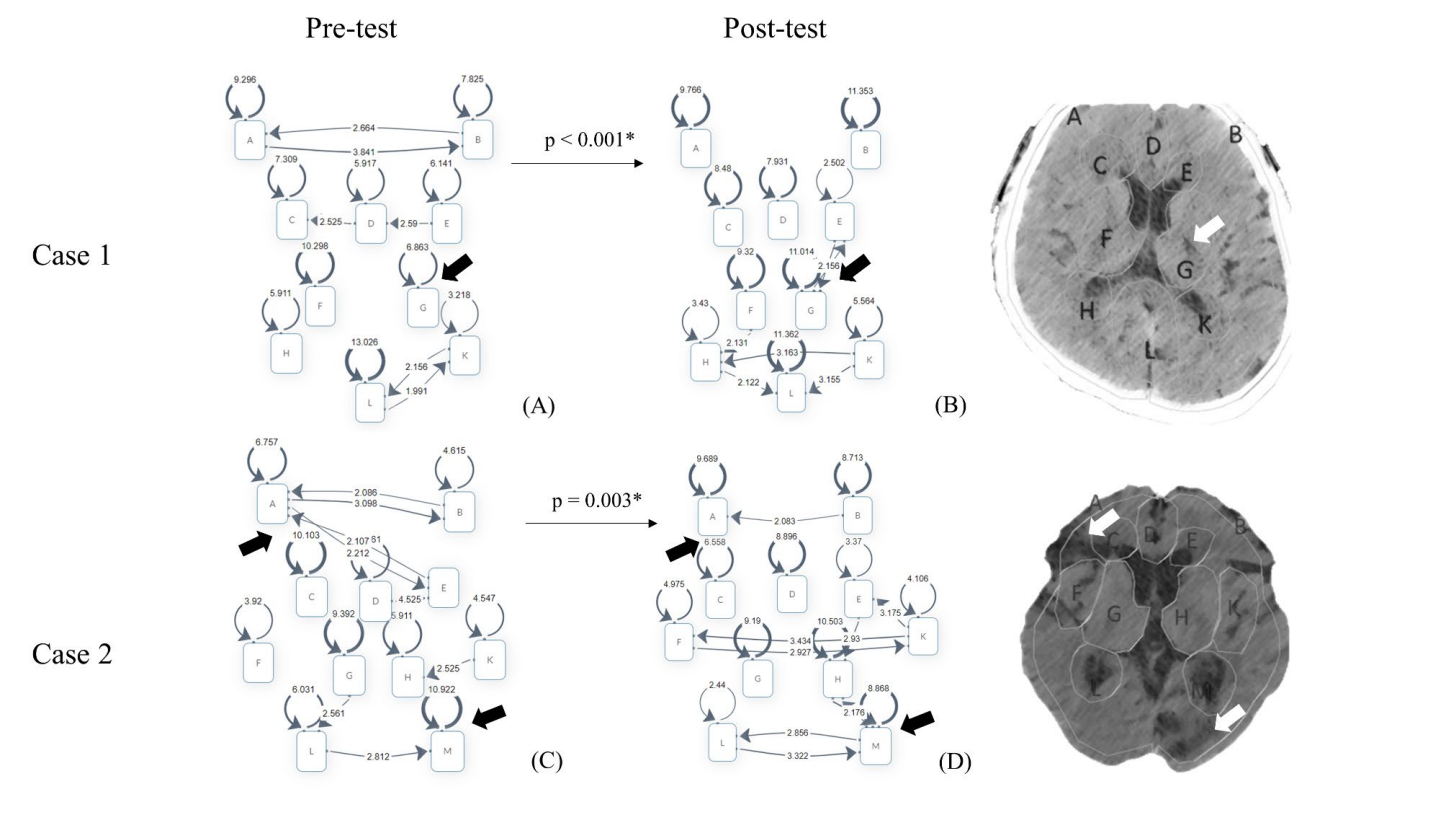
Lagged sequential analysis of the scan path before and after instruction The A-M codes in the left and middle columns represent the corresponding anatomical locations in the right column. The numerical values presented in these two figures show the Z score of each behaviour sequence. The larger the value of Z, the more pronounced the reading pattern is. In case 1, the image reading patterns of the medical students were localized at the upper portion of the image before instruction (A), but became more widespread circling the areas between bilateral hemispheres and left basal ganglia post-test (B). In case 2, the medical students’ reading behaviour showing a bilateral symmetric reading pattern was restrict to the upper and lower portions before instruction (C), but changed to the upper, middle, and lower portions post-test (D) ^#^Arrows in the figures indicate the target lesions

Regarding the competency in systematic image reading, the scores of systematic OP ratings were all improved in the both cases [Case 1: pre-test vs. post-test, 0 (0,1) vs. 1 (1,2), p < 0.001; Case 2: 0 (0,1) vs. 1 (1,2), p < 0.001] after examining by Wilcoxon signed-rank tests. With regards to the reading behaviour changes in eye-tracking study, the pre-test and post-test TEs, compared by using Wilcoxon signed-rank tests, were 3.42 ± 0.62 and 3.67 ± 0.37 respectively (p = 0.001) in case 1, and were 3.42 ± 0.76 and 3.75 ± 0.37, respectively in case 2 (p = 0.002; Table 2). The results of LMMs suggested a significant interaction between instruction and AOI, F (9, 1620) = 3.21, p < 0.001 for case 1; a significant interaction between instruction and AOI, F (9, 1458) = 2.75, p = 0.004 for case 2 (Table S4). The scan path, heat map, and LSA of the scan path before and after instruction are illustrated in Figs. 3 and 4. In addition, the median self-efficacy score regarding brain CT reading were 35.5 (29,41), and 38.5 (32,43) before and after the instruction (p = 0.08; Wilcoxon signed-rank test).

## Discussion

Our results demonstrated that improvements in the OP assessments may occur concurrently with behavioural changes in the brain CT reading sequences of the medical students, which provides some neuroscience evidence on the value of brain CT reading skill assessments. The development of a reliable and rigorous assessment tool is meaningful, as it will enable better evaluation of image reading learning, allow educators to objectively validate the results of their findings, and impact the cognitive exercise of learning medical image reading.

Although structured OP is an easy-to-use assessment tool in daily practice, it is often unclear whether the learners report their findings based on observations of important anatomical locations, or whether they just assume the findings and report orally without concordant reading behaviour. We evaluate the levels of systematic reading behaviours in terms of OP ratings, LSA, TE, and LMMs. First, the participants’ levels of systematic reading improved because OP ratings improved in both cases. Given reading behaviours could be explicitly observed by eye movement, LSA, TE, and LMMs were performed to interpret the systematic reading behaviours from the eye movement perspective. That is, the levels of systematic views of CT scans could be potentially evaluated in terms of LSA, LMMs, and TE. TE showed the variability of attentional swift among AOIs. The significant increasing of TE in both cases showed that participants may incline to shift attention among AOIs frequently after instruction. Furthermore, LSA could help us to identify significant attentional swift from a chronological perspective. With the help of LSA, we could know more about how the AOIs were visited in a chronological order. Third, the significant interactions of LMMs provide evidence indicating that dwell time on different ROIs changed after instruction. Specifically, dwell time for several ROIs increased after instruction. In sum, participants’ systematic OP could potentially reflected changes in the eye movement accordingly. Moreover, we observed the learners’ bilateral symmetric reading behaviour was restricted to the upper and lower portions before instruction, but was extended to the upper, middle, and lower portions after instruction based on fixation reports. In sum, our results showed that the reading pattern changed after instruction. Furthermore, we also noted a concurrent improvement in scores of systematic OP ratings. It is possible that the structured OP might reflect the learners’ reading behaviour. Further studies are needed to confirm whether this effect is long-lasting or whether it could be applied in various situations.

A previous study on pathology reading patterns demonstrated two types: (1) a scanning type of search, whereby the pathologists focused on many points within the image but only for a short moment; and (2) a selective type of search, whereby the pathologists limited their search to specific areas within the lesion [28]. Systematic viewing seems to improve the performance of learners [29]. Among those parameters in the eye-tracking evaluation, DT demonstrates that how long learners are likely to spend looking at the target lesions, FC shows the amount of fixation points within the area of interest. In this study, we used DT and FC to assess whether our learners get more or less attention on target lesions and both these may reflect the levels of cognitive demand needed when interpreting medical images [30]. We only found significant changes in the DTs on the target lesions for case 1 after instruction in our work. It is possible that multiple-lesion task could be more difficult than single-lesion task for novice learners. In addition, the accuracy of anatomical and lesion descriptions did not improve after instruction either. Previous studies have reported that experts may read brain images from the top down, indicating that they may view the images in a goal-oriented manner [31]. This goal-oriented, global-focal, holistic mechanism used by experts relies on identifying potentially relevant regions, pre-attentive filtering, and subsequent cognitive evaluation [9]. Previous eye-tracking study found that experts were faster to dwell on abnormalities and that they concentrated more on the surrounding area compared to novice learners [32]. It is possible the students’ prior knowledge of neurological diseases may be insufficient to allow for meaningful viewing and to give a correct answer. Our students also had similar self-efficacy and did not gain in confidence with regards to their brain CT reading skills after instruction. It is possible that more clinical experience or case logs and increased knowledge related to the specific disease or lesions are required to demonstrate improvements in these assessments [33].

Instruction materials and assessment tools should differ between experts and novice learners, and among the different stages of the learning process. For example, avoiding omission errors is essential for novice learners, while reducing heuristic and cognitive errors are more important for experienced learners [34, 35]. Omission errors while searching visually are a common source of medical image interpretation errors [35]. Structured assessments have been used for clinical competence [36], debriefing [37], and communication skills [38]. Thus, structured assessments can be applied in medical imaging education. Structured reporting has been proposed as a potential solution to improve the quality of radiology reports [39]. Disease-specific report templates may improve the clarity and quality of reports, while checklist style reports could reduce diagnostic errors, particularly in incidental findings [39]. Several studies have achieved consensus in structured medical imaging reporting [40, 41]. For novice learners, the learning curve in image reading may include a comprehensive reading of all anatomically important areas, knowing where to look [42], reducing interpretation failures leading to under- or over-diagnosis [43], handling multi-target lesion images [44], and reducing errors in non-serious lesions [44]. Our study showed the improvement of the systematic oral reporting performance and eye-tracking changes in TE were detected in these novice clinical learners after training, suggesting that improvement in structured reporting may be noted earlier than advances in diagnostic accuracy, and could be suitable for rating the response in novice learners.

This study had several limitations. First, the OP of each student was rated by a single rater. We did not achieve consensus with regards to the rating in an expert committee, this could be a source of personal prejudice. However, we coded the participants with an anonymous number, which may have helped to reduce bias. The eye-tracking technique also provided objective evidence supporting the difference in interpretation. Second, heterogeneity of the students’ learning style, experience of the students’ prerequisite capacities may also have confounded the results. We tried to enrol the same degree of medical student and recorded their previous clinical rotation status. Third, the contents of the instruction could also have influenced the results. We used the Delphi process to determine the teaching materials and to design the rating checklist, which could also have reduced bias from the instructional design based on personal preference. Fourth, the prior knowledge regarding sectional-anatomy of brain structures of these medical students may be different. A 2-hour workshop could be insufficient for these novice learners to be familiar with brain CT interpretation in a systematic way. A Ten-second duration might be too short for these novice learners to complete their image reading. All these could confound the results of OP and eye-tracking findings. Another reason might result from the complexity of TE and components of OP. Given TE is a general estimation of variability for multiple AOIs and components of OP are spoken words, the complex information could also make it difficult to evaluate the findings. Another limitation was that we only chose stroke cases, while in emergency department, head trauma is another common situation that clinicians face to interpret brain CT images, in which epidural/subdural hematoma or subarachnoid haemorrhage are commonly noted, thus could be included in future studies. Head injury after stroke is not uncommon, and these lesions are contraindication for intravenous thrombolytic therapy. This was another reason why we set “systematic image reading” as an initial goal for novice learners. In addition, this study was insufficient to demonstrate the casual relationship between eye-tracking behaviour and OP performance. Moreover, our data could also be insufficient to give a conclusive answer that the kind of “attentional shift” measured by LSA implies systematicity rather than simply less focus. Finally, all the students came from a single medical school, and so the generalizability of our results remains limited. Further studies are needed to define whether these changes are long lasting and whether they are as effective in post-graduate or neurology resident doctors.

## Conclusion

Our eye-tracking results showed that the improvements in the performance of OP may occur concurrently with behavioural changes of brain CT reading sequences. Our findings may guide the development of teaching materials and assessment tools in the future.

## Electronic supplementary material

Below is the link to the electronic supplementary material.

Supplementary Material 1

## Data Availability

The data that support the findings of this study are available from the corresponding author upon reasonable request.

## List of all the abbreviations

AOI: Area of interest
CT: Computed tomography
DT: Dwell time
FC: Fixation counts
LMM: Linear mixed models.
LSA: Lag sequential analysis.
OP: Oral presentation
TE: Transition entropy
