# Supplementary material for: Oral presentation assessment and image reading behaviour on brain computed tomography reading in novice clinical learners: an eye-tracking study
Source: BMC Med Educ. 2022 Oct 25;22:738. doi: 10.1186/s12909-022-03795-9 (PMC9597969; doi:10.1186/s12909-022-03795-9)

**Supplementary files**

**Table S1.** Checklist of systematic brain CT reading

| Reading bone windows | | | | | |  |  |  |
| --- | --- | --- | --- | --- | --- | --- | --- | --- |
| Sequential reading from extra-axial to intra-axial | | | | | | | |  |
| Identify the image symmetry of the bilateral hemispheres | | | | | | | | |
|  | Extra-axial lesions | | | | |  |  |  |
|  |  | Epidural and subdural space | | | | |  |  |
|  |  | Interhemispheric fissure | | | | |  |  |
|  |  | Sylvian fissure | | | |  |  |  |
|  |  | Ventricles | | |  |  |  |  |
|  |  |  | Lateral ventricles | | | |  |  |
|  |  |  | Temporal horns of lateral ventricles | | | | | |
|  | Intra-axial lesion | | | | |  |  |  |
|  |  | ACA territory | | | |  |  |  |
|  |  | PCA territory | | | |  |  |  |
|  |  | MCA territory, basal ganglia and thalamus | | | | | | |
|  |  | Border zone areas | | | |  |  |  |
|  |  |  | | MCA-ACA border zone | | |  |  |
|  |  |  | | MCA-PCA border zone | | |  |  |
|  |  | Temporal lobes | | | |  |  |  |
|  |  | Mass effect, mid-line shift, or herniation | | | | | |  |
| CT, computerized tomography; ACA, anterior cerebral artery; MCA, middle cerebral artery; PCA, posterior cerebral artery. | | | | | | | | |

| **Table S2.** Operating definitions of oral presentation grades | |
| --- | --- |
| Trial 1 | |
| 0 | Disorganized and unable to interpret systematically, and unable to answer correctly left internal capsule/putamen lesion |
| 1 | Could only partially and systematically interpret images of non-lesional sites, and could not correctly answer left internal capsule/putamen lesion |
| 2 | Could systematically interpret images of non-lesional sites, but could not correctly answer left internal capsule/putamen lesion |
| 3 | Able to answer left internal capsule/putamen lesion correctly, but could only partially and systematically interpret images of non-lesional sites |
| 4 | Able to correctly answer left internal capsule/putamen lesion, and could systematically interpret images of non-lesional sites |
| Trial 2 | |
| 0 | Disordered and unable to interpret systematically, and unable to answer one of left occipital lesion and right frontal lesion |
| 1 | Able to correctly answer one of left occipital lesion and right frontal lesion, or only partially and systematically interpret images of non-lesional sites |
| 2 | Could answer one of left occipital lesion and right frontal lesion correctly, but could systematically interpret the images of non-lesional sites |
| 3 | Able to correctly answer left occipital lesion and right frontal lesion, but could only partially and systematically interpret non-lesional images |
| 4 | Able to correctly answer left occipital lesion and right frontal lesion, and could systematically interpret images of non-lesional sites |
| ACA, anterior cerebral artery; MCA, middle cerebral artery; PCA, posterior cerebral artery. | |

| **Table S3**. Content of self-efficacy questionnaire | |
| --- | --- |
| SE1 | I believe I can get an excellent grade in the stroke CT interpretation instruction. |
| SE2 | I believe I can understand the most difficult parts of the stroke CT interpretation instruction. |
| SE3 | I am confident that I can learn the basic concepts taught in the stroke CT interpretation instruction. |
| SE4 | I am confident that I can understand the most complex parts of the stroke CT interpretation instruction. |
| SE5 | I am confident that I can do well in the content specified in the stroke CT interpretation instruction, and that I can pass the exam well. |
| SE6 | I expect to get high marks in the stroke CT interpretation course. |
| SE7 | I am confident that I am proficient in the methodological skills taught in the stroke CT interpretation course. |
| SE8 | Considering the difficulty of stroke CT interpretation, the ability of the teacher, and my own, I think I can learn stroke CT interpretation well. |

| **Table S4.** Test statistics and average dwell time of the linear mixed model | | | | | |
| --- | --- | --- | --- | --- | --- |
| Case 1 | | | Case 2 | | |
| effects | F | p values | effects | F | p values |
| Instruction (I) | 0.91 | 0.34 | Instruction (I) | 6.17 | 0.01** |
| AOI | 20.74 | <0.001*** | AOI | 22.13 | <0.001*** |
| I*AOI | 3.21 | <0.001*** | I*AOI | 2.75 | 0.004** |
|  |  |  |  |  |  |
| AOI | Pre DT | Post DT | AOI | Pre DT | Post DT |
| A | 108.04 | 187.89** | A | 179.81 | 229.79* |
| B | 98.30 | 214.33*** | B | 49.70 | 135.90*** |
| C | 125.12 | 159.50 | C | 185.70 | 163.61 |
| D | 204.89 | 186.99 | D | 228.01 | 239.96 |
| E | 63.90 | 76.80 | E | 156.65 | 128.59 |
| F | 239.33 | 221.33 | F | 209.36 | 177.44 |
| G | 240.48 | 214.19 | G | 174.51 | 194.55 |
| H | 72.03 | 79.91 | K | 99.39 | 136.38 |
| K | 115.96 | 94.86 | L | 62.56 | 108.07* |
| L | 322.03 | 247.48* | M | 217.34 | 257.03 |

^＃^**p* < 0.05; ***p* < 0.01; ****p* < 0.001

A, B, C, D, E, F, G, H, K, L, M represent AOIs in case 1 and 2.

AOI, area of interest; DT, dwell time.

**Figure S1**

Figure showing the protocol of the eye-tracking study.


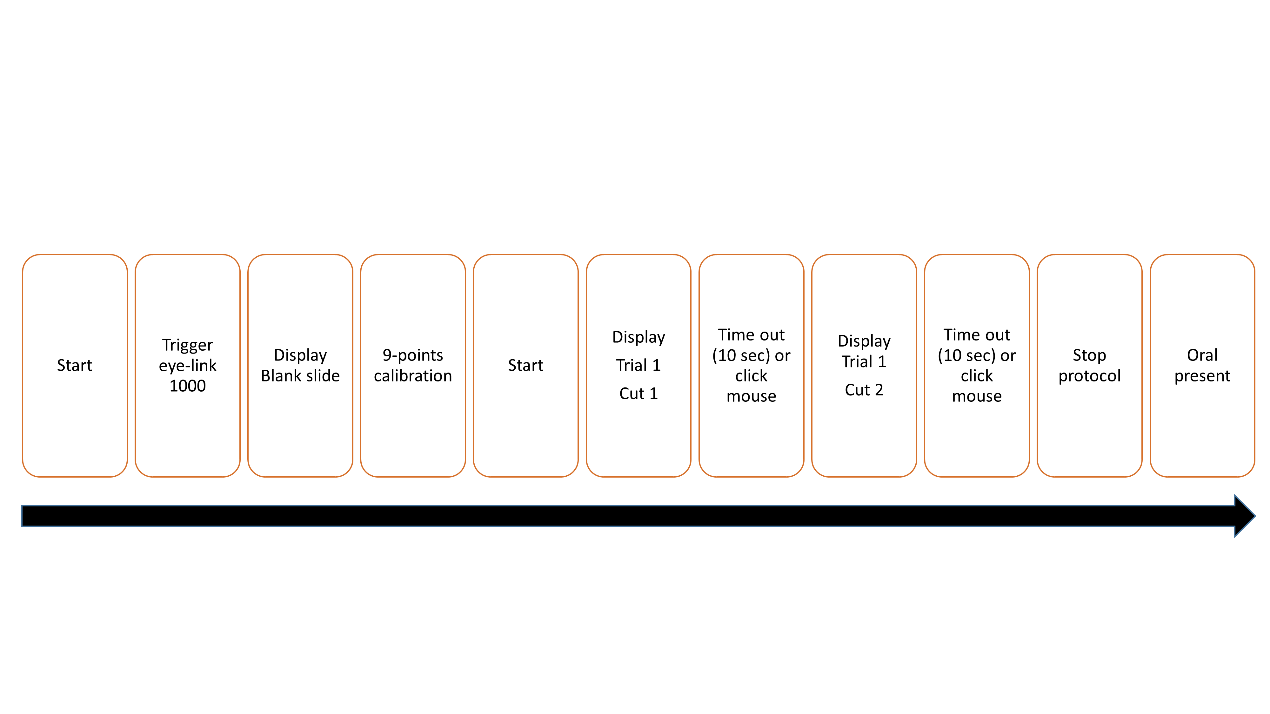

Supplement: Supplementary file 1 — Supplementary Material 1 [file 12909_2022_3795_MOESM1_ESM.docx]
